# Relationship between antibody avidity, Fc-mediated functional activity and longevity of malaria vaccine responses in clinical trials

**DOI:** 10.3389/fimmu.2026.1808822

**Published:** 2026-04-07

**Authors:** Jessica L Horton, Jo-Anne Chan, Liriye Kurtovic, Linda Reiling, Gaoqian Feng, Kristina E M Persson, Jahit Sacarlal, Robin F Anders, James S McCarthy, Carlota Dobaño, Michelle J Boyle, James G Beeson

**Affiliations:** 1 Burnet Institute, Melbourne, VIC, Australia; 2 Department of Medicine and Department of Infectious Diseases University of Melbourne, Melbourne, VIC, Australia; 3 School of Translational Medicine, Monash University, Melbourne, VIC, Australia; 4 National Vaccine Innovation Platform, NHC Key Laboratory of Antibody Technique, Jiangsu Key Laboratory of Pathogen Biology, Department of Pathogen Biology, Nanjing Medical University, Nanjing, China; 5 Department of Laboratory Medicine, Lund University, Lund, Sweden; 6 Clinical Chemistry and Pharmacology, Laboratory Medicine, Skåne University Hospital, Lund, Sweden; 7 Centro de Investigaco em Saude de Manhica, Maputo, Mozambique; 8 Faculdade de Medicina, Universidade Eduardo Mondlane (UEM), Maputo, Mozambique; 9 Department of Biochemistry and Genetics, La Trobe University, Melbourne, VIC, Australia; 10 Victorian Infectious Diseases Services, Doherty Institute, University of Melbourne, Melbourne, VIC, Australia; 11 ISGlobal, Hospital Clınic Universitat de Barcelona, Barcelona, Catalonia, Spain

**Keywords:** affinity, children, complement, Fc-mediated antibody functions, malaria, phagocytosis, vaccines

## Abstract

**Background:**

Advancing the development of highly efficacious and long-lasting malaria vaccines is hampered by incomplete knowledge of protective immune mechanisms and an absence of established correlates of immunity. Antibody avidity is frequently evaluated as an indicator of a high-quality or protective antibody response to vaccination. However, the importance of avidity in vaccine-induced immunity to malaria remains unclear.

**Methods/approach:**

We investigated the association between antibody avidity, Fc-mediated functional activities, and antibody longevity in two different *Plasmodium falciparum* vaccine trials; a phase 1 trial of merozoite surface protein 2 in malaria-naïve adults, and a phase 2b trial of the RTS,S vaccine in African children.

**Results:**

In both trials, we found that the magnitude of IgG induced by vaccination correlated strongly with antibody avidity indicating the two vaccine immunogenicity outcomes are related. Antibody avidity was not a major determinant of Fc-mediated functional activity, including complement fixation, binding of Fcγ-receptors, or opsonic phagocytosis. In analysis models, IgG magnitude was a stronger determinant of function than IgG avidity. Although avidity positively correlated with antibody functional activities, this was largely due to the confounding effect of the correlation between IgG magnitude and Fc functional activity. Additionally, we found that antibody avidity showed only a weak and antigen-specific association with antibody longevity. Similar findings in the two vaccine trials, which differed by vaccine antigen, adjuvant, and age group, suggests that these observations are likely to be generalisable and not specific to a vaccine type.

**Conclusion:**

Antibody avidity was not a better predictor of Fc-mediated functional activity than antibody magnitude and avidity was not a strong correlate of antibody longevity. These findings suggest that vaccine approaches that focus on increasing antibody avidity *per se* may not be sufficient to achieve more efficacious or long-lasting vaccines.

## Introduction

Malaria remains a major global health threat and progress in combating malaria has stalled. Highly protective and long-lasting vaccines are needed to reduce the global burden and achieve progress towards malaria elimination (1). To date, leading malaria vaccine candidates have not demonstrated potent and/or long-lasting protection in clinical trials (2–4). The RTS,S vaccine, the first malaria vaccine recommended by the World Health Organization, is now being implemented in young children (<2y.o.) in Africa and acts against *Plasmodium falciparum*, the species responsible for most malaria disease (5). It has modest efficacy, which wanes markedly within 12–18 months (2). RTS,S, and the R21 vaccine based on the same antigenic construct, target the sporozoite stage of *P. falciparum*, which is the form inoculated by a feeding mosquito. Other vaccines in development are based on antigens of blood-stage merozoites; while some candidates have shown promising results, no blood-stage vaccine candidates have achieved higher efficacy than RTS,S and R21, or completed phase 3 trials (1, 6, 7). Advancing the development of highly efficacious and long-lasting malaria vaccines is impaired by incomplete knowledge of protective immune mechanisms and an absence of established markers of immunity. While antibodies are vital in malaria immunity, a high antibody titre does not guarantee protection following vaccination. Increasing evidence indicates that specific functional properties are important for immunity and may be better correlates of immunity than IgG titre (8, 9).

One measure of quality or protective potential of vaccine-induced antibodies is avidity, which describes the overall strength of a binding interaction between an antibody and antigen. Affinity is the strength of a single binding site on an antibody to a single epitope on an antigen. Since antibodies typically have multiple binding sites, and antigens have multiple epitopes, avidity represents the cumulative binding strength of all these interactions together (10). In a developing immune response, B cells undergo somatic hypermutation in their immunoglobulin genes. B cells that produce antibodies with higher affinity for an antigen are generally preferentially selected to proliferate. Therefore, the overall affinity and avidity of the IgG pool may increase over time or after multiple vaccinations. Because of its potential importance, avidity is frequently evaluated as an indication of a high-quality antibody response to vaccination. However, the value of high avidity antibodies in vaccine-induced immunity to malaria remains unclear. In clinical trials of RTS,S, high avidity of antibodies induced to the vaccine antigen (circumsporozoite protein; CSP) has shown some association with vaccine efficacy in children (11) (12). However, there is little data on how avidity may enhance antibody protective functional activities and support malaria immunity. Antibodies can contribute to immunity by directly inhibiting host cell invasion (by sporozoites or merozoites) and through Fc-mediated functions that include fixing serum complement to induce the complement cascade or binding Fc-receptors on immune cells to trigger opsonic phagocytosis by neutrophils, monocytes, and macrophages, and antibody-dependent cytotoxicity by natural killer cells (13–18). For RTS,S, antibody Fc-mediated functions have been correlated with protection (9, 19) (20).

Achieving long-lasting immunity is a major challenge for malaria vaccines; therefore, biomarkers of immune longevity would be valuable in vaccine development and evaluation. Antibody avidity may be a marker of long-lasting antibody immunity if it reflects affinity maturation of antibodies and B cell memory. As long-lived plasma cells are thought to mature from B cells following germinal centre reactions including affinity maturation (21, 22), antibody avidity may correlate with the presence of these long-lasting antibody-secreting cells.

We investigated antibody avidity and its association with protective Fc-mediated functions and antibody longevity in clinical trials of two malaria vaccines, which differed by antigen, adjuvant, and age group, factors which may influence antibody responses. We studied a Phase 2b trial of the RTS,S vaccine, which targets the major surface protein of *P. falciparum* sporozoites (23), conducted in young African children (24). The second was a Phase 1 trial evaluating safety and immunogenicity of a vaccine targeting a major protein on the *P. falciparum* merozoite surface, merozoite surface protein 2 (MSP2), in malaria-naïve adults (25). A previous trial of a 3-antigen vaccine, which included MSP2, demonstrated substantial strain-specific efficacy mediated by MSP2 vaccine responses (26). Additionally, MSP2 is a significant target of naturally-acquired immunity (27, 28). For both vaccines, antibody Fc-mediated functional activities are important mechanisms of immunity (9, 14).

## Methods

### Malaria vaccine cohorts

Samples were obtained from two completed malaria vaccine clinical trials: a Phase 1a trial of a dual isoform MSP2 vaccine in malaria-naïve adults (25)(Australian New Zealand Clinical Trials Registry 12607000552482), and a Phase 2b trial of RTS,S in children aged 1–4 years in malaria-endemic regions of Mozambique (24)(NCT00197041). In the MSP2 vaccine trial, 23 malaria-naïve Australian adults received at least two doses of vaccine containing the 3D7 and FC27 isoforms of MSP2 (with Montanide ISA-720 adjuvant) at 12-week intervals. Serum samples collected prior to vaccination (day 0), and at 112 and 168 days after first vaccination were tested. Peak IgG response following vaccination occurred around day 112 in all participants, and 96% of subjects were classified as positive of IgG at day 112 (25). RTS,S is based on the CSP antigen and is formulated with AS01/AS02 as the adjuvant (a mixture of QS21 and MPLA). For analysis of RTS,S vaccine responses, 50 participants with serum samples available three months and eight and a half months after the first dose were randomly selected from a cohort of young children enrolled in a Phase 2b trial of RTS,S/AS02A in Mozambique. In that trial, children received 3 vaccines doses at 1-month intervals (24, 29); 97% of children were regarded as seropositive for IgG to the NANP-repeat antigen post vaccination (9). Sera from malaria-naïve adult Australian donors were used to establish a baseline of non-specific antibody binding as pre-vaccination samples were unavailable. Ethics approval was obtained from the Human Research Ethics Committees of the Queensland Institute of Medical Research and Alfred Health (for the MSP2 vaccine trial) and from Mozambican National Health and Bioethics Committee, Hospital Clınic of Barcelona Ethics Committee, PATH Research Ethics Committee, and Alfred Health Human Research and Ethics Committee (for the RTS,S trial). All participants or their guardians provided written informed consent.

### Antibody responses

Vaccine-specific IgG responses were assessed using established enzyme-linked immunosorbent assays (ELISAs) (30). Briefly, wells were coated with antigen (recombinant MSP2 3D7, MSP2 FC27, CSP, NANP repeat region of CSP or C-terminal region of CSP) (0.5 μg/mL) diluted in phosphate-buffered saline (PBS) overnight at 4 °C. Non-specific binding was minimised by blocking wells with 1% casein (weight/volume in PBS) for 2 hours at 37 °C. Diluted, heat-inactivated (56 °C for 45 minutes) serum samples were added for a 2 hour incubation. Samples were tested in duplicate.

To assess avidity, wells were treated with either NH_4_SCN (2M and 3M for the MSP2 and RTS,S vaccine cohorts, respectively) or 0.1% casein (w/v) for 20 minutes. Antigen-specific IgG was detected with anti-human IgG conjugated to horseradish-peroxidase (HRP) and followed by the addition of 2,2’-azino-bis(3-ethylbenzothiazoline-6-sulphonic acid) (ABTS) (Life Technologies, USA) or tetramethylbenzidine (TMB) (Life Technologies) as an enzymatic substrate. The principle of this assay is that antibodies are eluted with increasing concentrations of the chaotrope (ammonium thocyanate) enabling antibody avidity to be estimated. The concentration of ammonium thiocyanate was optimised for assays using CSP or MSP as the antigen. Optical density was measured at 450 nm for TMB and 405 nm for ABTS. Wells were washed three times prior to the addition of a new reagent, and incubations were performed at room temperature unless otherwise specified. Samples were tested in duplicate.

Fc-mediated antibody functions were measured as above with previously described modifications to measure antibody binding to C1q (13, 30), the initial step of the classical complement cascade, or to FcγRIIa and FcγRIIIa dimers (15, 31). Phagocytosis of antibody-opsonised *P. falciparum* merozoites by THP1 monocytes was performed as previously described (16) (32). Detailed protocols are available here: https://protocolexchange.researchsquare.com/article/pex-2510/v1 https://protocolexchange.researchsquare.com/article/pex-2509/v1. Samples were tested in duplicate

For the MSP2 vaccine cohort, dissociation rates of antibodies from MSP2 3D7 were also assessed with surface plasmon resonance (SPR) and biolayer interferometry (BLI) approaches; it was not possible to do this with pediatric samples from the RTS,S trial because of insufficient sample volume. For SPR, plasma samples from 17 participants were measured on the Biocore 3000, using methods that have been previously evaluated and optimized with MSP2 protein coupled to the surface of SPR chips (33, 34). Samples were diluted in running buffer at two or more dilutions to confirm obtained k_d_ values were independent of concentration. Due to non-specific binding when testing serum samples in BLI analysis (evident in high levels of binding to both antigen-coated and reference biosensors), total IgG was purified from serum samples of 22 participants using the Melon Gel IgG Spin Purification Kit (Thermo Fisher Scientific) and concentrated with Amicon Ultra-0.5 Centrifugal Filter Devices (Millipore). IgG dissociation was measured with the OctetRED96 (FortéBio, USA) using the Amine Reactive Second-Generation Reagent Kit (FortéBio). Biosensors were bound to MSP2 3D7 (10 μg/mL in pH 5 acetate buffer) or bovine serum albumin (BSA; 5% in acetate buffer) for reference biosensors. To regenerate biosensors, with the goal of removing any residual bound antibodies, biosensors were washed three times, each involving exposure to 10 mM glycine-HCl (pH 1.5) for 30 seconds followed by a 10 second wash in kinetics buffer. Biosensors were regenerated a maximum of three times (allowing four cycles of sample testing). Each sample was tested twice, with the order of samples randomised for each assay to minimise the effect of cycle number (i.e., the number of times biosensors were regenerated prior to sample binding) on binding kinetics. Background signal, measured with reference biosensors, was subtracted from sample values and the mean of the dissociation rates (k_d_) from each assay calculated.

### Statistical analysis

In the MSP2 vaccine trial, day 0 samples formed a baseline reading for vaccine-induced IgG. In the RTS,S cohort, given there was malaria exposure prior to vaccination, a positivity threshold was established using the upper 95% confidence limit of the mean OD of Melbourne donor samples without NH_4_SCN exposure. Avidity was measured as an avidity index (AI), calculated as the percentage of antigen-specific IgG remaining following NH_4_SCN treatment [(IgG with NH_4_SCN)/(IgG with 0.1% casein)%]. Associations between avidity measures, IgG magnitude and antibody Fc-mediated functions or maintenance were assessed with Spearman’s correlation in GraphPad Prism. Significant associations were explored in multivariate linear regression analysis in RStudio (35). Due to non-linear relationships, antibody function and maintenance data were first log_2_-transformed; in datasets with negative values (i.e. after subtraction of negative control wells) a constant value (< 0.1 OD) was added to all samples to obtain a positive dataset and enable log-transformation.

## Results

### IgG avidity and magnitude are related in vaccine responses

**Figure 1:**
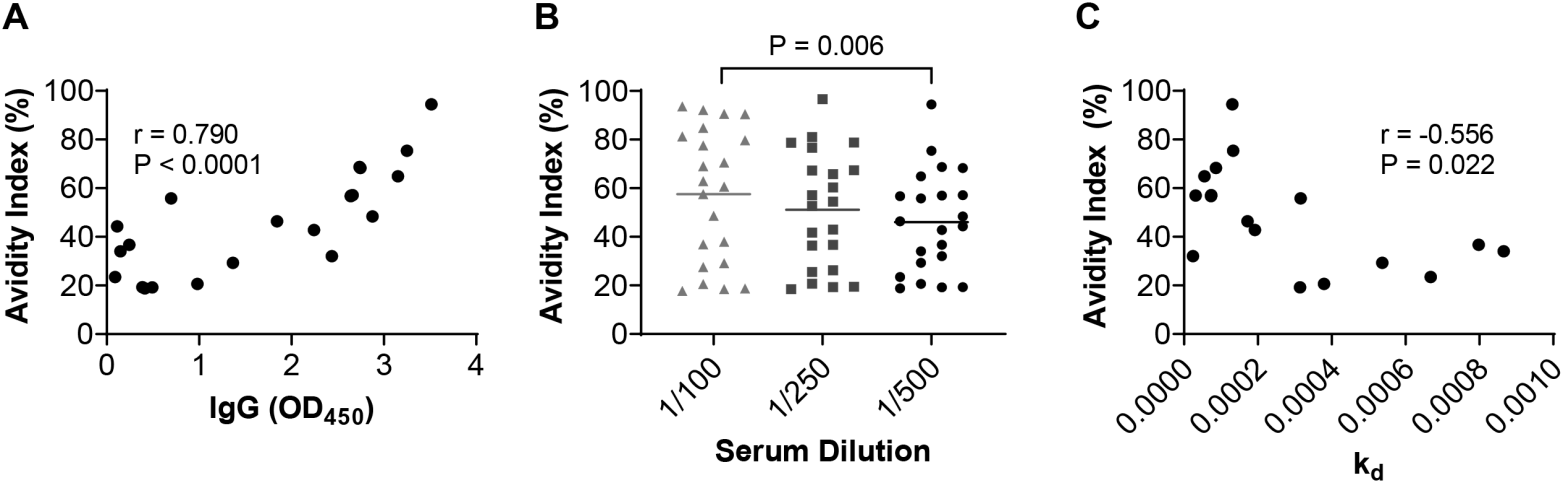
AI values measured in the MSP2 vaccine cohort against MSP2-3D7 relative to antibody concentration. **(A)** AI values showed a significant positive association with IgG magnitude. **(B)** AIs declined when samples were tested at decreasing serum concentrations (Friedman test). **(C)** AIs showed a moderate negative association with dissociation rates (k_d_) determined using SPR; lower k_d_ indicates higher affinity. Spearman’s correlation coefficients and p values are shown. Antibody parameters were measured among samples collected at day 112 of the study.

We first evaluated IgG avidity and magnitude in a phase 1 trial of the MSP2 vaccine in adults where larger sample volumes allowed us to explore different methodologies. The vaccine was based on a mixture of the two major isoforms of MSP2, known as 3D7 and FC27. We expected some correlation between avidity and IgG magnitude given that IgG production and affinity maturation may be occurring concurrently in these malaria-naïve participants following vaccination. Different methods to evaluate antibody avidity in human vaccine trials have been reported in the literature. Chaotrope-based avidity ELISAs, where serum antibodies are exposed to a chaotrope (frequently thiocyanate or urea) to elute weakly bound antibodies from their target, have been the most widely reported method for quantifying antibody avidity. Using this approach, using samples collected post vaccination (day 112), we found that avidity index (AI) values had a strong positive correlation with IgG magnitude against the MSP2-3D7 antigen used in the vaccine (**Figure 1A**; r=0.79, p<0.001). AIs were highest when serum samples were tested at a 1/100 dilution and decreased significantly with serum concentrations, demonstrating that AI measures are affected by antibody concentration in the assay (**Figure 1B**). We also quantified AI of antibodies to the alternative isoform of MSP2-FCR27, which was also included in the vaccine. AI values for MSP2-FC27 also strongly correlated with IgG magnitude (r=0.816, p<0.0001) and displayed a moderate and significant association with affinity measured using SPR (r=0.569, p=0.019; **Supplementary Figure S1**). In the Phase 2b RTS,S vaccine trial in young children, we analysed samples after completion of the 3-dose vaccination schedule (Month 3). We found that there was also a strong correlation between antigen-specific AI and IgG (r = 0.833, P < 0.0001; **Supplementary Figure S2**). Overall, these results indicate that the correlation between the IgG magnitude and avidity of vaccine responses is observed for different vaccine types and age groups.

To complement these data, we also evaluated avidity using SPR (measured as dissociation constant, k_d_), which measures avidity independent of antibody concentration (33, 34), using samples collected at day 112 from the MSP2 vaccine trial. Data from the two assays were significantly correlated. AI values from avidity ELISAs demonstrated a significant negative correlation with k_d_ values (**Figure 1C**; r=-0.556, p=0.022); in SPR, higher k_d_ reflects more rapid dissociation and therefore lower avidity. The k_d_ values were also highly correlated with IgG magnitude (r = -0.782, P = 0.0004), suggesting that the observed correlation between AI and IgG is a true biological observation and not simply a technical factor of ELISA-based avidity quantification. However, limitations of SPR include is low throughput, need for specialised equipment, and the higher sample volume requirement for assays. Because of the low sample volume from the paediatric RTS,S trial, we could not measure avidity using SPR for that study. BLI is an additional method that has been used for estimating antibody avidity in malaria vaccine responses (36). Therefore, we also explored this method of evaluating avidity of IgG against MSP2-3D7. We found this assay, performed with antigen bound to amine reactive biosensors, was poorly suited to measuring avidity with polyclonal antibodies from samples as optimal regeneration of biosensors could not be achieved. To reduce potential systematic error from the cycle number on dissociation rate measurements, biosensors were regenerated a maximum of three times, and purified IgG from each sample was tested in two separate experiments, with the sample order randomised before each assay. The mean dissociation rate quantified using BLI showed a moderate positive correlation with the dissociation rate measured by SPR (r = 0.593, P = 0.014) and a moderate negative correlation with AI (r = -0.363, P = 0.097). Given the wide use of chaotrope-based ELISAs for measuring avidity, and their suitability for high throughput and small sample volumes (as typical in paediatric studies), we used data from this platform for addressing our subsequent research aims.

### Avidity has minimal association with Fc-mediated antibody functions for MSP2 vaccine responses

**Figure 2:**
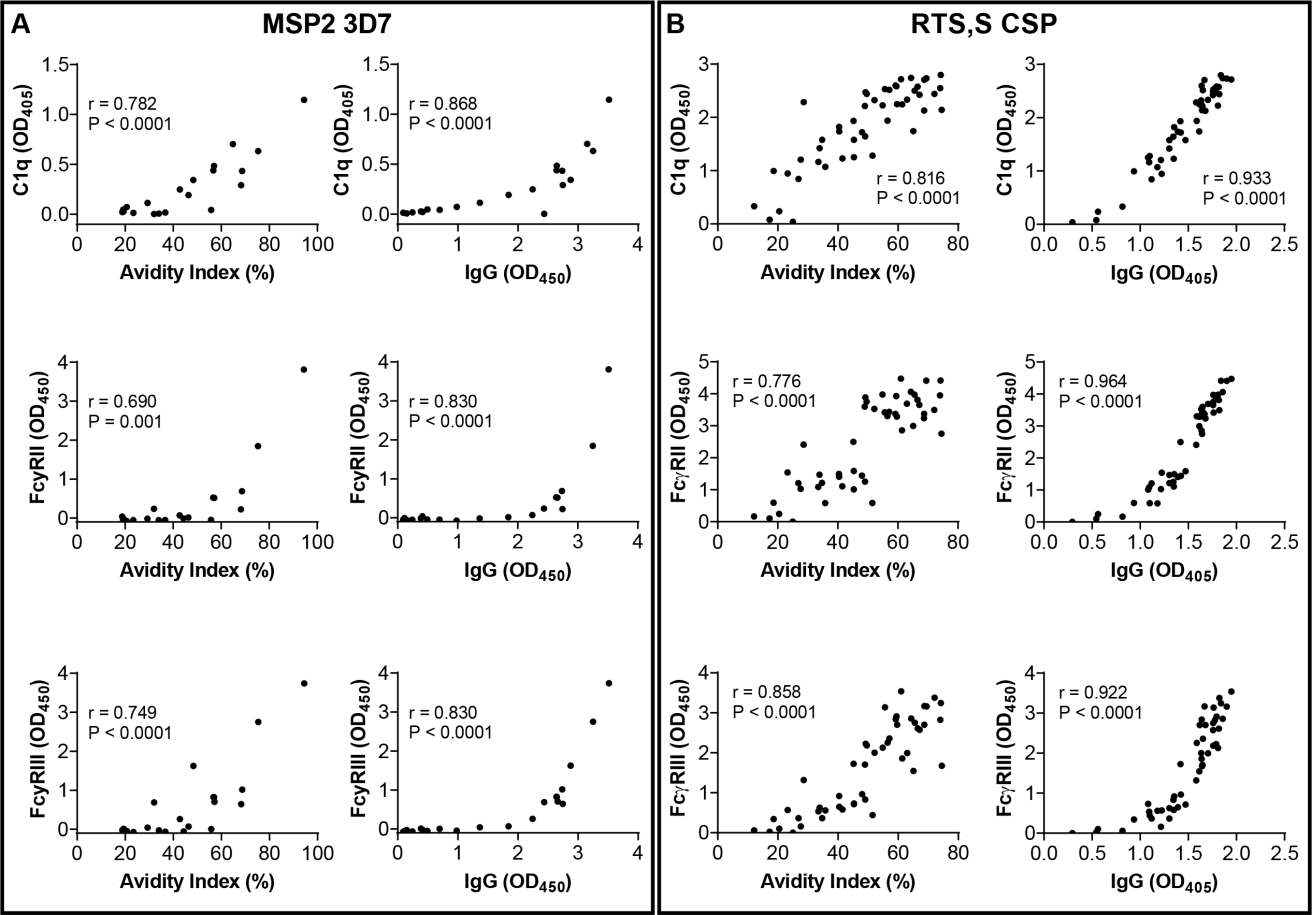
Relationships between Fc-mediated antibody binding to C1q and FcγRs with avidity and IgG magnitude. Association of C1q, FcγRIIa and FcγRIII binding with IgG avidity (AI) or IgG magnitude in the MSP2 vaccine cohort (using MSP2-3D7 antigen) **(A)**, and in the RTS,S cohort against full-length CSP **(B)**. Spearman’s correlation coefficient and P-value are shown. Data for MSP2-FC27 are shown in **Supplementary Figure S3**. Antibody parameters were measured among samples collected at day 112 for the MSP2 vaccine and month 3 for the RTS,S vaccine.

Antibodies mediate several effector functions via interactions with their Fc region, including binding C1q to initiate the classical complement cascade and binding Fc-receptors on immune cells; these functional activities have been correlated with protective immunity (9, 13, 16, 17, 29, 30, 32). In the MSP2 vaccine trial, both IgG magnitude and avidity displayed strong and significant positive associations with C1q-fixation against MSP2-3D7 (r=0.868 and r=0.782, p<0.001, respectively) (**Figure 2A**; using samples collected at day 112). We next considered the associations with C1q-fixation for both IgG avidity and magnitude in a log_2_-transformed linear regression model, which accounted for the potential effect of antibody concentration on IgG avidity measurements. This model estimated that for each 1-unit (OD_450_) increase in anti-MSP2 (3D7) IgG, C1q-fixation increased by 2.0-fold (P = 0.07). In the model, each 1% increase in AI had minimal effect on C1q-fixation (back-transformed coefficient 1.03) and was not statistically significant (P = 0.14). These associations were similar when we evaluated antibodies specific to the MSP2-FC27 isoform in the analysis model. There were strong positive correlations between C1q and each of IgG and AI (r=0.938 for IgG and r=0.808 for AI; **Supplementary Figure S3**). However, only IgG magnitude was a significant predictor of C1q binding in the regression model (P < 0.0001), whereas avidity had a minimal and non-significant effect (**Table 1**).

**Table 1:** Effects of AI and IgG magnitude on Fc-mediated antibody binding to C1q, FcγRIIa (CD32a) and FcγRIII (CD16) in linear regression models.

Cohort	Antigen	IgG			Avidity index			Model		
		*Coefficient* *[95% CI]*	*Coefficient* *(back-* *transformed)*	*P-value*	*Coefficient* *[95% CI]*	*Coefficient* *(back-* *transformed)*	*P-value*	*Adj. R²*	*F (df)*	*P-value* *(model)*
C1q-fixation										
MSP2 vaccine	MSP2 (3D7)	0.965 [-0.079, 2.009]	1.952	0.068	0.042 [-0.016, 0.100]	1.030	0.143	0.618	16.36 (2,17)	**0.0001**
	MSP2 (FC27)	1.557 [1.125, 1.988]	2.942	**< 0.0001**	-0.007 [-0.035, 0.020]	0.995	0.580	0.898	88.57 (2,18)	**< 0.0001**
RTS,S	CSP	3.505 [2.792, 4.218]	11.354	**< 0.0001**	-0.013 [-0.028, 0.003]	0.991	0.108	0.836	120.5 (2,45)	**< 0.0001**
	NANP	5.405 [3.331, 7.479]	42.366	**< 0.0001**	0.049 [ 0.008, 0.090]	1.034	0.021	0.537	26.52 (2,42)	**< 0.0001**
	C-terminal	4.849 [3.110, 6.588]	28.814	**< 0.0001**	0.025 [-0.021, 0.070]	1.017	0.287	0.638	38.92 (2,41)	**< 0.0001**
FcγRIIa-binding										
MSP2 vaccine	MSP2 (3D7)	1.214 [0.427, 2.002]	2.321	**0.005**	0.035 [-0.008, 0.079]	1.025	0.105	0.760	29.48 (2,16)	**< 0.0001**
RTS,S	CSP	5.968 [4.716, 7.219]	62.583	**< 0.0001**	-0.0320 [-0.060, -0.004]	0.978	0.024	0.806	98.53 (2,45)	**< 0.0001**
	NANP	9.176 [7.453, 10.899]	578.583	**< 0.0001**	0.023 [-0.011, 0.057]	1.016	0.180	0.779	78.32 (2,42)	**< 0.0001**
FcγRIII-binding										
MSP2 vaccine	MSP2 (3D7)	1.988 [1.546, 2.430]	3.967	**< 0.0001**	0.007 [-0.018, 0.033]	1.005	0.545	0.925	123.6 (2,18)	**< 0.0001**
RTS,S	CSP	5.449 [4.266, 6.633]	43.695	**< 0.0001**	-0.003 [-0.029, 0.023]	0.998	0.805	0.850	134.3 (2,45)	**< 0.0001**
	NANP	6.950 [5.493, 8.406]	123.624	**< 0.0001**	0.003 [-0.026, 0.031]	1.002	0.860	0.718	56.94 (2,42)	**< 0.0001**

Functional data were log_2_-transformed due to non-linear relationships. Results for each model reported as coefficient for 1-unit increase in predictor (1 OD IgG or 1% AI), coefficient after correction for log_2_-transformation and P-value for each predictor variable, and adjusted R^2^, F-statistic (with degrees of freedom) and P-value of F-statistic. Significant (p < 0.05) relationships between IgG or AI and function highlighted in bold text. Antibody parameters were measured among samples collected at day 112 for the MSP2 vaccine and month 3 for the RTS,S vaccine.

Similarly, binding of FcγRIIa (CD32a) and FcγRIII (CD16) by IgG specific to MSP2-3D7 was somewhat more strongly correlated with IgG magnitude than avidity (**Figure 2A**). In linear regression models only IgG had a significant association with FcγR-binding; a 1-unit increase in IgG was associated with 2.3-fold and 4.0-fold increases in FcγRIIa and FcγRIII, respectively. In contrast, AI had no discernible effects on FcγR binding activity (**Table 1**). As FcγRIIa and FcγRIII mediate opsonic phagocytosis, we also considered possible associations between antibody avidity and phagocytosis of merozoites (expressing the MSP2-FC27 isoform) by monocytes *in vitro.* Phagocytosis showed no association with AI measures (r = 0.032, P = 0.887), and a moderate association with IgG magnitude (r = 0.373, P = 0.087).

### IgG magnitude, rather than avidity, is the main determinant of functional activity among RTS,S vaccinated children

Among the RTS,S vaccine trial samples (collected at month 3), we investigated antibody binding to the full-length CSP antigen and the epitope regions of CSP that are included in the RTS,S vaccine (NANP-repeat and C-terminal regions). C1q-fixation by antibodies consistently showed a stronger correlation with IgG magnitude than with avidity using full length CSP or the NANP-repeat and C-terminal regions (**Figure 2B**; **Supplementary Figure S4**). In regression models, only IgG magnitude was a significant predictor of C1q-fixation to either full-length CSP or the C-terminal region of CSP (**Table 1**). For antibodies specific to the NANP-repeat region of CSP, IgG magnitude was the primary predictor of C1q-binding, and AI showed a significant but weaker correlation. For every 1-unit (OD_405_) increase in anti-NANP IgG, the model predicted C1q binding to increase over 40-fold (P < 0.0001); equivalently, for every 0.1 OD_405_ increase in IgG, a 1.5-fold increase in C1q binding was predicted. AI showed a comparatively minor effect, with a 10% increase in AI required for a similar 1.4-fold C1q increase (P = 0.021).

Binding of FcγRIIa and FcγRIII by vaccine-induced IgG showed stronger correlations with IgG magnitude than with avidity. Only IgG magnitude had a significant and positive association with FcγR-binding in linear regression models. For FcγRIIa, each 0.1 OD_405_ increase in IgG was associated with a 1.5-fold rise in FcγRIIa binding (P < 0.0001). Conversely, AI showed a weak negative trend whereby a 10% AI increase was associated with a 20% decline in FcγRIIa binding (P = 0.024). There was no association between FcγRIII-binding and AI, whereas IgG magnitude showed a strong and significant association with FcγRIII binding (**Table 1**). Similarly, only magnitude of anti-NANP IgG was a significant predictor of FcγR-binding (**Table 1**). Few samples showed FcγR-binding by antibodies targeting the C-terminal region of CSP so these were not assessed in regression models, but somewhat stronger correlations were evident with IgG magnitude than with avidity (**Supplementary Figure S4**).

### Avidity shows some antigen-specific associations with antibody maintenance

We investigated whether IgG avidity was related to longevity of vaccine antibody responses. To assess the temporal decline of vaccine-induced IgG in each vaccine cohort, antibody maintenance was measured as the percentage of the peak IgG response retained at the follow-up visit for each participant.

**Figure 3:**
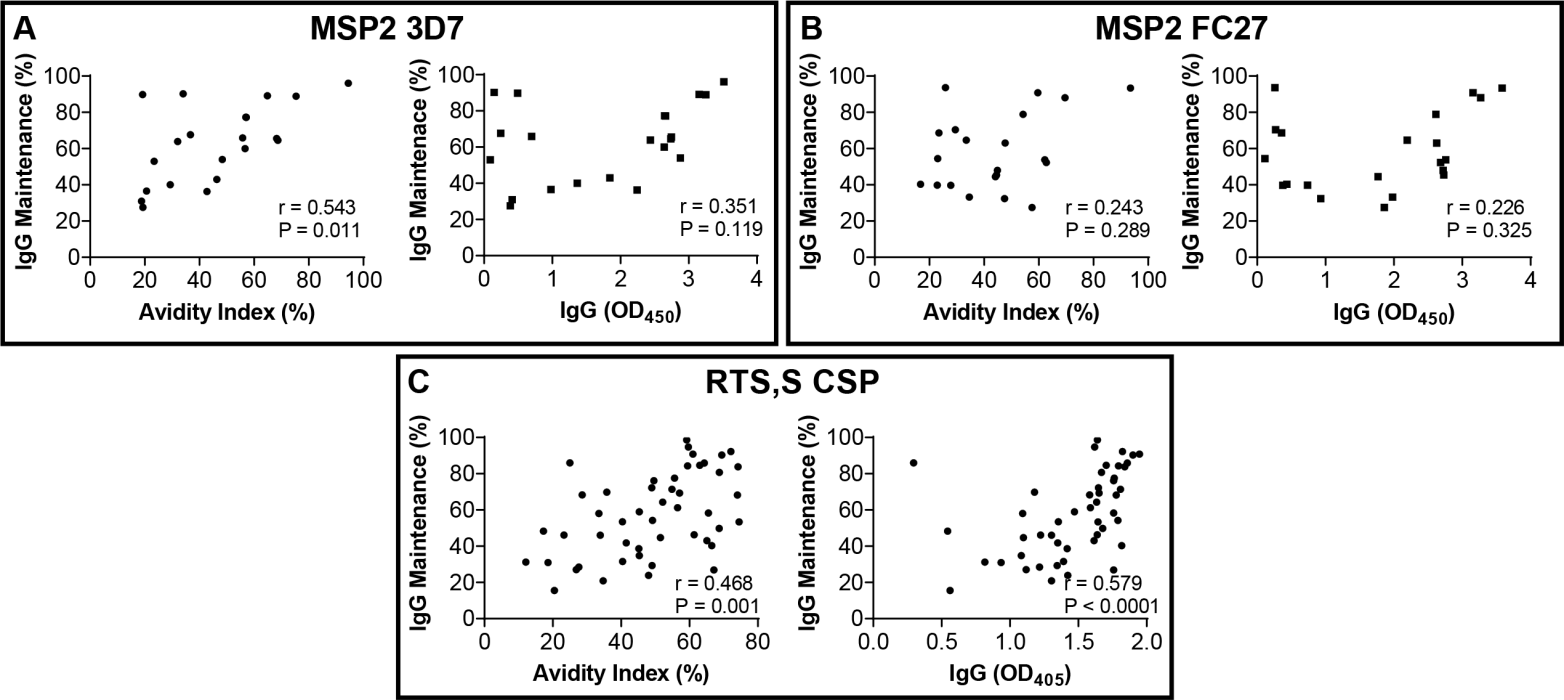
Relationships between IgG maintenance and avidity or IgG magnitude against specific vaccine antigens. **(A)** Maintenance of IgG against MSP2-3D7 in the Phase 1 MSP2 vaccine cohort, measured as (IgG at day168)/(IgG at day 112)%, showed a moderate correlation with avidity but not with IgG magnitude at month 3. **(B)** Maintenance of IgG against MSP2-FC27, measured as (IgG at day168)/(IgG at day 112)%, was associated with neither avidity nor IgG magnitude. **(C)** In the RTS,S cohort, maintenance of anti-CSP IgG, measured as (IgG at month 3)/(IgG at month 8.5)%, showed a positive association with both avidity and IgG magnitude. Spearman’s correlation coefficient and P-value shown.

In the MSP2 vaccine cohort, IgG against both 3D7 and FC27 forms of MSP2 increased following the first dose and reached a peak response around day 112 in most participants (25). The proportion of peak IgG lost over follow-up, measured at day 168, varied greatly between individuals and a single participant who showed a small increase in IgG over time was excluded from analysis. Maintenance of IgG to MSP2-3D7 displayed a moderate and significant correlation with avidity (r=0.543, p=0.011) but there was no significant correlation with IgG magnitude (measured at day 112) (**Figure 3A**). In a linear regression model, only AI was a significant predictor of IgG maintenance, with each 1% increase in avidity associated with a 1.02-times increase in IgG maintenance, equivalent to a 1.2-fold increase with every additional 10% increase in AI (P = 0.017). However, a low R^2^ (adjusted) of 0.320 in the regression model suggests a large portion of variation in IgG maintenance is not explained by avidity (**Table 2**). In contrast, for MSP2-FC27 there was no clear relationship between IgG maintenance and either AI or IgG magnitude (**Figure 3B**).

**Table 2:** Effects of AI and IgG magnitude on IgG maintenance (%) in linear regression models.

Cohort	Antigen	IgG			Avidity index			Model		
		*Coefficient* *[95% CI]*	*Coefficient* *(back-* *transformed)*	*P-value*	*Coefficient* *[95% CI]*	*Coefficient* *(back-* *transformed)*	*P-value*	*Adj. R²*	*F (df)*	*P-value* *(model)*
MSP2 vaccine	MSP2 (3D7)	-0.137 [-0.445, 0.170]	0.909	0.361	0.022 [0.004, 0.039]	1.015	**0.017**	0.320	5.703 (2,18)	**0.012**
RTS,S	CSP	0.461 [-0.346, 1.267]	1.376	0.256	0.011 [-0.007, 0.028]	1.007	0.235	0.235	8.219 (2,45)	**< 0.001**
RTS,S	NANP	1.330 [0.550, 2.109]	2.514	**0.001**	-0.005 [-0.020, 0.010]	0.997	0.507	0.250	8.008 (2,40)	**0.001**
RTS,S	CT	0.218 [-0.297, 0.733]	1.163	0.398	0.007 [-0.007, 0.0201]	1.005	0.338	0.071	2.651 (2,41)	0.083

IgG maintenance results, measured as the percentage of the peak IgG response present at follow-up in each vaccine cohort, were log_2_-transformed due to non-linear relationships. Results for each model reported as coefficient for 1-unit increase in predictor (1 OD IgG or 1% AI), coefficient after correction for log_2_-transformation and P-value for each predictor variable, with adjusted R^2^, F-statistic (with degrees of freedom) and P-value of F-statistic. Significant (p < 0.05) relationships between IgG or AI and function highlighted in bold text.

In the RTS,S trial, the magnitude of vaccine-specific IgG generally declined substantially at 6 months after the 3^rd^ and final vaccine dose, compared to 1 month after completion of the 3-dose vaccine schedule. For full-length CSP, IgG maintenance (month 8 IgG/month 3 IgG as %) showed moderately strong correlations with both AI and IgG titre (r=0.468 and r=0.579, respectively; p<0.01) (**Figure 3C**). However, neither was significantly associated with antibody maintenance in a linear regression model (**Table 2**), suggesting other factors have a major role in influencing longevity. For anti-NANP IgG, following exclusion of two participants who showed peak IgG responses at month 8, IgG magnitude showed a strong positive correlation with IgG maintenance (**Supplementary Figure S5**) and was a significant predictor in regression analysis, while avidity had no effect (**Table 2**). For IgG to the CSP C-terminal region, both IgG avidity and magnitude showed a weak positive correlation with maintenance (**Supplementary Figure S5**), but in regression analysis neither were significant determinants of IgG maintenance (**Table 2**).

## Discussion

Antibody avidity or affinity has been evaluated in malaria vaccine trials, but the relevance of avidity to protective immunity remains undefined. We investigated the association between antibody avidity and protective antibody properties, including Fc-mediated functional activities and longevity, in two different malaria vaccine trials. In both trials, we found that the magnitude of IgG induced by vaccination correlated strongly with antibody avidity indicating the two vaccine outcomes are related. However, we found that high avidity antibodies did not drive enhanced Fc-mediated functional activity; in analysis models, IgG magnitude was a stronger determinant of function than IgG avidity. Although avidity correlated with the magnitude of antibody functions, this was largely due to the confounding effect of the correlation between IgG magnitude and Fc functional activity. Additionally, we found that antibody avidity showed only a weak and inconsistent association with antibody longevity. A strength of our study is that we evaluated responses in two vaccine trials that differed by vaccine antigen, adjuvant, and age group. Despite these differences, the key findings were very similar, suggesting our conclusions are likely to be generalisable and not specific to a vaccine type.

IgG magnitude was the key driver of antibody functional activities, whereas avidity was not consistently associated with antibody function in regression analyses. Both RTS,S and MSP2 vaccines induced antibodies that had complement fixation and Fc-receptor binding activity, and IgG magnitude correlated with these functional activities with both vaccines. Avidity was not a significant predictor of C1q-fixation in the MSP2 vaccine cohort. In the RTS,S cohort, avidity showed a weak positive association with C1q-fixation by antibodies targeting the NANP repeat region but not full-length CSP or the C-terminal region, highlighting that avidity is not a consistent marker of functional activity across vaccine targets. Similarly, avidity was not a predictor of FcγRIII-binding in either the MSP2 or RTS,S vaccine cohorts, and displayed contradictory effects on FcγRII-binding, despite strong correlations between AI measurements and FcγR-binding. We had hypothesised that high avidity antibodies may enhance interactions with FcγRs, and therefore effector cell activity. Binding of C1q and the receptors FcγRII and FcγRIII requires clustering of IgG bound to the antigen (37, 38). Higher avidity might provide more stable and compact IgG complexes that better promote binding of C1q and FcγRs. However, we found no correlation of antibody avidity with Fc-mediated function and opsonic phagocytosis of merozoites *in vitro* for MSP2, and weak or no associations for RTS,S. Our findings may indicate that Fc-mediated functional activity has a threshold of avidity required for antibody functions, and higher avidity above that gives little benefit. The IgG avidity induced by these vaccines may be above this threshold. We focused on antibody Fc-functions because they are important in antibody mediated immunity targeting merozoite surface antigens and in RTS,S vaccine immunity (9, 17, 29) (13, 16, 30, 32). While IgG is a better predictor of functional activity, it is possible that avidity still plays a role in optimal protective functions *in vivo*. Antibody avidity may have different associations with functions or protective immunity in naturally-acquired immunity (34, 39, 40). Our data raises questions whether quantifying avidity adds major value above the quantification of IgG magnitude or antibody functions when evaluating vaccine immunity. A prior study of RTS,S in infants and young children found that IgG avidity to the C-terminal region was associated with protection (11). Future studies evaluating antibody responses, functions and avidity in vaccines trials among different age groups would also be informative. A high avidity monoclonal antibody to CSP has shown efficacy in protection against malaria in clinical trials (41). However, the importance of IgG avidity protective function is not currently clear. Antibodies can also contribute to immunity by direct inhibition of host-cell invasion, and the relationship between this antibody function and avidity could be addressed in future studies. Some data using different monoclonal antibodies targeting the same or similar epitopes suggests that avidity may be associated with better inhibition of sporozoite invasion (42). Direct antibody inhibition of merozoite invasion, or growth inhibition of blood-stage *P. falciparum*, *in vitro* has not been consistently associated with protective immunity (43). Data on the associations between sporozoite invasion inhibition and vaccine efficacy are limited, but this function has not been associated with protection in vaccine trials (1) (44).

Poorly sustained immunity and short-lived antibodies induced by malaria vaccines is an enduring challenge and there is a strong need for predictive markers of long-term immune responses to inform vaccine development and trials (1). Long lived plasma cells, which secrete antibodies and survive for years, usually exhibit greater binding affinity than memory B cells and extrafollicular plasma cells (21, 22, 45). Therefore, we had hypothesised that high avidity antibodies may reflect a better sustained antibody response. Of the five antigen constructs tested, avidity was a significant, but not strong, predictor of antibody maintenance for MSP2-3D7 only. Avidity was not associated with maintenance of antibodies to MSP2-FC27. This discrepancy between isoforms may result from the substantial amino acid sequence differences between the 3D7 and FC27 forms of MSP2 (46). IgG magnitude was also not consistently associated with antibody maintenance for RTS,S vaccine response, showing a positive and significant association only for IgG to the NANP repeat region. Our findings suggest there may be an antigen-specific association between avidity and IgG durability, but avidity cannot be defined as a marker for the induction of a sustained antibody response. Moreover, our analyses suggest that factors other than IgG magnitude and avidity are major determinants or predictors of longevity. Further research is needed to identify these factors and identify parameters that may be predictive of longevity.

Here we quantified antibody avidity, which represents the combined strength of multiple binding interactions between an antibody molecule and an antigen. Chaotrope-based avidity ELISAs, where serum antibodies are exposed to a chaotrope (frequently thiocyanate or urea) to strip weakly bound antibodies from their target, are the most common approach used to measuring antibody avidity due to their affordability and accessibility, but they can be affected by antibody concentration (10). This was evident in our studies with the MSP2 vaccine cohort. However, AI showed a moderately strong correlation with antibody dissociation-rate as measured by an alternative method using SPR, suggesting avidity ELISA measurements are a valid estimate of antibody binding strength if antibody concentration is considered in analysis. SPR and BLI technologies are frequently used to quantify the affinity of antibody Fab regions or monoclonal antibodies, but more recently they have been applied to polyclonal samples to measure avidity to malaria antigens (36, 47). However, few malaria studies have adopted these techniques due to the increased time, expertise and expenses involved. We found limitations in adapting BLI to measure polyclonal antibody avidity in serum samples. Specifically, additional sample purification steps were required and biosensor regeneration was incomplete, substantially increasing the cost of the assay. It is likely chaotrope-based ELISAs will remain prevalent, but our analyses show that it is important to evaluate IgG magnitude and avidity together when assessing associations between avidity and study outcomes. We also acknowledge that many published papers from analysis of human serum samples use the term affinity when the term avidity would be more precise because IgG, IgA, and IgM, the most abundant antibodies in serum, all have multiple epitope binding sites.

In conclusion, these studies provide important new data on the relevance of antibody avidity in malaria vaccine immunity. Our findings show that following vaccination antibody avidity is associated with the antibody response magnitude, such that high magnitude IgG responses typically also have high avidity. IgG magnitude is a better predictor of Fc-mediated functional activity than avidity. Furthermore, neither avidity nor IgG magnitude was a valuable correlate of the induction of a sustained antibody response. Our findings suggest that other antibody characteristics should be prioritised when assessing the quality of antibody responses induced by malaria vaccines. Vaccine approaches that focus on increasing antibody avidity *per se* may not achieve more efficacious or long-lasting vaccines.

## Data Availability

The raw data supporting the conclusions of this article will be made available by the authors, without undue reservation.
