# Supplementary material for: Relationship between antibody avidity, Fc-mediated functional activity and longevity of malaria vaccine responses in clinical trials
Source: Front Immunol. 2026 Apr 7;17:1808822. doi: 10.3389/fimmu.2026.1808822 (PMC13095604; doi:10.3389/fimmu.2026.1808822)
Supplement: Supplementary file 1 [file Supplementaryfile1.docx]

**Relationship between antibody avidity, Fc-mediated functional activity and longevity of malaria vaccine responses in clinical trials**

Jessica L Horton^1,2^, Jo-Anne Chan^1,3^, Liriye Kurtovic^1,3^, Linda Reiling^1.3^, Gaoqian Feng, ^1,4^ Kristina E M Persson^5,6^, Jahit Sacarlal^8,9^, Robin F Anders^10^, James S McCarthy^11^, Carlota Dobaño^7^, Michelle J Boyle^1,2^ & James G Beeson^1,2,3^

## Supplementary Data

**Supplementary Figure S1:** **Avidity and IgG against MSP2-FC27 in the MSP2 vaccine cohort. A)** AI values showed a moderately strong and significant positive association with IgG magnitude. **B)** AIs declined when samples were tested at decreasing serum concentrations. **C)** AIs showed a moderate negative association with dissociation rates (k_d_) calculated with SPR. Antibody parameters were measured among samples collected at day 112.

**Supplementary Figure S2: Correlations between avidity and IgG against CSP in the RTS,S cohort. A.** The association between AI and IgG was strongest at M3, **B.** but still showed a moderate correlation at M8 when IgG values were lower. Spearman’s correlation coefficients and P-values are shown.

**Supplementary Figure S3: In the MSP2 vaccine cohort, C1q-fixation by IgG to MSP2-FC27 was positively correlated avidity and IgG magnitude.** Spearman’s correlation coefficient and P-value shown. Antibody parameters were measured among samples collected at day 112.

**Supplementary Figure S4: Fc-mediated binding to C1q and FcγRs by antibodies to the NANP (A) and C-terminal (B) regions of CSP in the RTS,S cohort.** Spearman’s correlation coefficient and P-value shown. Antibody parameters were measured among samples collected at day 112 for the MSP2 vaccine and month 3 for the RTS,S vaccine.

**Supplementary Figure S5: Association of IgG maintenance in the RTS,S cohort, when measured against the NANP (A) or C-terminal (B) regions of CSP, with avidity and IgG magnitude.** Spearman’s correlation coefficient and P-value shown.

1. Beeson JG, Kurtovic L, Dobano C, Opi DH, Chan JA, Feng G, Good MF, Reiling L, Boyle MJ. Challenges and strategies for developing efficacious and long-lasting malaria vaccines. Sci Transl Med. 2019 Jan 9;11(474). Epub 2019/01/11. doi:10.1126/scitranslmed.aau1458. Cited in: Pubmed; PMID 30626712.

2. RTS S Clinical Trials Partnership. Efficacy and safety of RTS,S/AS01 malaria vaccine with or without a booster dose in infants and children in Africa: final results of a phase 3, individually randomised, controlled trial. The Lancet. 2015;386(9988):31-45. eng. Epub 2015/04/23. doi:10.1016/S0140-6736(15)60721-8. Cited in: Pubmed; PMID 25913272.

3. White MT, Verity R, Griffin JT, Asante KP, Owusu-Agyei S, Greenwood B, Drakeley C, Gesase S, Lusingu J, Ansong D, Adjei S, Agbenyega T, Ogutu B, Otieno L, Otieno W, Agnandji ST, Lell B, Kremsner P, Hoffman I, Martinson F, Kamthunzu P, Tinto H, Valea I, Sorgho H, Oneko M, Otieno K, Hamel MJ, Salim N, Mtoro A, Abdulla S, Aide P, Sacarlal J, Aponte JJ, Njuguna P, Marsh K, Bejon P, Riley EM, Ghani AC. Immunogenicity of the RTS,S/AS01 malaria vaccine and implications for duration of vaccine efficacy: secondary analysis of data from a phase 3 randomised controlled trial. The Lancet Infectious Diseases. 2015 2015/12/01/;15(12):1450-1458. doi:<https://doi.org/10.1016/S1473-3099(15)00239-X>.

4. Datoo MS, Dicko A, Tinto H, Ouédraogo J-B, Hamaluba M, Olotu A, Beaumont E, Ramos Lopez F, Natama HM, Weston S, Chemba M, Compaore YD, Issiaka D, Salou D, Some AM, Omenda S, Lawrie A, Bejon P, Rao H, Chandramohan D, Roberts R, Bharati S, Stockdale L, Gairola S, Greenwood BM, Ewer KJ, Bradley J, Kulkarni PS, Shaligram U, Hill AVS, Mahamar A, Sanogo K, Sidibe Y, Diarra K, Samassekou M, Attaher O, Tapily A, Diallo M, Dicko OM, Kaya M, Maguiraga SO, Sankare Y, Yalcouye H, Diarra S, Niambele SM, Thera I, Sagara I, Sylla M, Dolo A, Misidai N, Simando S, Msami H, Juma O, Gutapaka N, Paul R, Mswata S, Sasamalo I, Johaness K, Sultan M, Alexander A, Kimaro I, Lwanga K, Mtungwe M, Khamis K, Rugarabam L, Kalinga W, Mohammed M, Kamange J, Msangi J, Mwaijande B, Mtaka I, Mhapa M, Mlaganile T, Mbaga T, Yerbanga RS, Samtouma W, Sienou AA, Kabre Z, Ouedraogo WJM, Yarbanga GAB, Zongo I, Savadogo H, Sanon J, Compaore J, Kere I, Yoni FL, Sanre TM, Ouattara SB, Provstgaard-Morys S, Woods D, Snow RW, Amek N, Ngetsa CJ, Ochola-Oyier LI, Musyoki J, Munene M, Mumba N, Adetifa UJ, Muiruri CM, Mwawaka JS, Mwaganyuma MH, Ndichu MN, Weya JO, Njogu K, Grant J, Webster J, Lakhkar A, Ido NFA, Traore O, Tahita MC, Bonko MdA, Rouamba T, Ouedraogo DF, Soma R, Millogo A, Ouedraogo E, Sorgho F, Konate F, Valea I. Safety and efficacy of malaria vaccine candidate R21/Matrix-M in African children: a multicentre, double-blind, randomised, phase 3 trial. The Lancet. 2024;403(10426):533-544. doi:10.1016/S0140-6736(23)02511-4.

5. World Health Organization. World malaria report 2025. Available from: <https://www.who.int/publications/i/item/9789240117822>. 2025.

6. Kurtovic L, Reiling L, Opi DH, Beeson JG. Recent clinical trials inform the future for malaria vaccines. Commun Med (Lond). 2021;1:26. Epub 2022/05/24. doi:10.1038/s43856-021-00030-2. Cited in: Pubmed; PMID 35602185.

7. Natama HM, Salkeld J, Some A, Soremekun S, Diallo S, Traore O, Rouamba T, Ouedraogo F, Ouedraogo E, Dabone KCS, Kone NA, Compaore ZMJ, Kafando M, Bonko MDA, Konate F, Sorgho H, Nielsen CM, Pipini D, Diouf A, King LDW, Shaligram U, Long CA, Cho JS, Lawrie AM, Skinner K, Roberts R, Miura K, Bradley J, Silk SE, Draper SJ, Tinto H, Minassian AM. Safety and efficacy of the blood-stage malaria vaccine RH5.1/Matrix-M in Burkina Faso: interim results of a double-blind, randomised, controlled, phase 2b trial in children. Lancet Infect Dis. 2024 Dec 10. Epub 2024/12/14. doi:10.1016/S1473-3099(24)00752-7. Cited in: Pubmed; PMID 39672183.

8. Opi DH, Kurtovic L, Chan J-A, Horton JL, Feng G, Beeson JG. Multi-functional antibody profiling for malaria vaccine development and evaluation. Expert Review of Vaccines. 2021;20(10):1257-1272.

9. Kurtovic L, Feng G, Hysa A, Haghiri A, O’Flaherty K, Wines BD, Santano R, D’Andrea L, Drummer HE, Hogarth PM. Antibody mechanisms of protection against malaria in RTS, S-vaccinated children: a post-hoc serological analysis of phase 2 trial. The Lancet Microbe. 2024;5(10).

10. Dimitrov JD, Lacroix-Desmazes S, Kaveri SV. Important parameters for evaluation of antibody avidity by immunosorbent assay. Analytical Biochemistry. 2011 2011/11/01/;418(1):149-151. doi:<https://doi.org/10.1016/j.ab.2011.07.007>.

11. Dobaño C, Sanz H, Sorgho H, Dosoo D, Mpina M, Ubillos I, Aguilar R, Ford T, Díez-Padrisa N, Williams NA, Ayestaran A, Traore O, Nhabomba AJ, Jairoce C, Waitumbi J, Agnandji ST, Kariuki S, Abdulla S, Aponte JJ, Mordmüller B, Asante KP, Owusu-Agyei S, Tinto H, Campo JJ, Moncunill G, Gyan B, Valim C, Daubenberger C. Concentration and avidity of antibodies to different circumsporozoite epitopes correlate with RTS,S/AS01E malaria vaccine efficacy. Nature Communications. 2019 2019/05/15;10(1):2174. doi:10.1038/s41467-019-10195-z.

12. Ajua A, Lell B, Agnandji ST, Asante KP, Owusu-Agyei S, Mwangoka G, Mpina M, Salim N, Tanner M, Abdulla S, Vekemans J, Jongert E, Lievens M, Cambron P, Ockenhouse CF, Kremsner PG, Mordmüller B. The effect of immunization schedule with the malaria vaccine candidate RTS,S/AS01E on protective efficacy and anti-circumsporozoite protein antibody avidity in African infants. Malaria Journal. 2015 2015/02/13;14(1):72. doi:10.1186/s12936-015-0605-7.

13. Boyle MJ, Reiling L, Feng G, Langer C, Osier Faith H, Aspeling-Jones H, Cheng Yik S, Stubbs J, Tetteh Kevin KA, Conway David J, McCarthy James S, Muller I, Marsh K, Anders Robin F, Beeson James G. Human Antibodies Fix Complement to Inhibit *Plasmodium falciparum* Invasion of Erythrocytes and Are Associated with Protection against Malaria. Immunity. 2015 2015/03/17/;42(3):580-590. doi:<https://doi.org/10.1016/j.immuni.2015.02.012>.

14. Kurtovic L, Behet MC, Feng G, Reiling L, Chelimo K, Dent AE, Mueller I, Kazura JW, Sauerwein RW, Fowkes FJI, Beeson JG. Human antibodies activate complement against *Plasmodium falciparum* sporozoites, and are associated with protection against malaria in children. BMC Medicine. 2018 2018/04/30;16(1):61. doi:10.1186/s12916-018-1054-2.

15. Feng G, Wines BD, Kurtovic L, Chan J-A, Boeuf P, Mollard V, Cozijnsen A, Drew DR, Center RJ, Marshall DL, Chishimba S, McFadden GI, Dent AE, Chelimo K, Boyle MJ, Kazura JW, Hogarth PM, Beeson JG. Mechanisms and targets of Fcγ-receptor mediated immunity to malaria sporozoites. Nature Communications. 2021 2021/03/19;12(1):1742. doi:10.1038/s41467-021-21998-4.

16. Osier FHA, Feng G, Boyle MJ, Langer C, Zhou J, Richards JS, McCallum FJ, Reiling L, Jaworowski A, Anders RF, Marsh K, Beeson JG. Opsonic phagocytosis of *Plasmodium falciparum* merozoites: mechanism in human immunity and a correlate of protection against malaria. BMC Medicine. 2014 2014/07/01;12(1):108. doi:10.1186/1741-7015-12-108.

17. Hill DL, Eriksson EM, Li Wai Suen CS, Chiu CY, Ryg-Cornejo V, Robinson LJ, Siba PM, Mueller I, Hansen DS, Schofield L. Opsonising antibodies to *P. falciparum* merozoites associated with immunity to clinical malaria. PLOS ONE. 2013;8(9):e74627.

18. Odera DO, Tuju J, Mwai K, Nkumama IN, Fürle K, Chege T, Kimathi R, Diehl S, Musasia FK, Rosenkranz M. Anti-merozoite antibodies induce natural killer cell effector function and are associated with immunity against malaria. Science Translational Medicine. 2023;15(682):eabn5993.

19. Kurtovic L, Atre T, Feng G, Wines BD, Chan J-A, Boyle MJ, Drew DR, Hogarth PM, Fowkes FJ, Bergmann-Leitner ES, Beeson JG. Multifunctional antibodies are induced by the RTS, S malaria vaccine and associated with protection in a phase 1/2a trial. The Journal of infectious diseases. 2021;224(7):1128-1138.

20. Suscovich TJ, Fallon JK, Das J, Demas AR, Crain J, Linde CH, Michell A, Natarajan H, Arevalo C, Broge T, Linnekin T, Kulkarni V, Lu R, Slein MD, Luedemann C, Marquette M, March S, Weiner J, Gregory S, Coccia M, Flores-Garcia Y, Zavala F, Ackerman ME, Bergmann-Leitner E, Hendriks J, Sadoff J, Dutta S, Bhatia SN, Lauffenburger DA, Jongert E, Wille-Reece U, Alter G. Mapping functional humoral correlates of protection against malaria challenge following RTS,S/AS01 vaccination. Sci Transl Med. 2020 Jul 22;12(553). Epub 2020/07/29. doi:10.1126/scitranslmed.abb4757. Cited in: Pubmed; PMID 32718991.

21. Suan D, Sundling C, Brink R. Plasma cell and memory B cell differentiation from the germinal center. Current Opinion in Immunology. 2017;45:97-102.

22. Nguyen DC, Joyner CJ, Sanz I, Lee FE-H. Factors Affecting Early Antibody Secreting Cell Maturation Into Long-Lived Plasma Cells [Review]. Frontiers in Immunology. 2019 2019-September-11;10:2138. English. doi:10.3389/fimmu.2019.02138.

23. Beeson JG, Kurtovic L, Valim C, Asante KP, Boyle MJ, Mathanga D, Dobano C, Moncunill G. The RTS,S malaria vaccine: Current impact and foundation for the future. Science Translational Medicine. 2022 Nov 16;14(671):eabo6646. eng. Epub 2022/11/17. doi:10.1126/scitranslmed.abo6646. Cited in: Pubmed; PMID 36383682.

24. Alonso PL, Sacarlal J, Aponte JJ, Leach A, Macete E, Milman J, Mandomando I, Spiessens B, Guinovart C, Espasa M, Bassat Q, Aide P, Ofori-Anyinam O, Navia MM, Corachan S, Ceuppens M, Dubois M-C, Demoitié M-A, Dubovsky F, Menéndez C, Tornieporth N, Ripley Ballou W, Thompson R, Cohen J. Efficacy of the RTS,S/AS02A vaccine against *Plasmodium falciparum* infection and disease in young African children: randomised controlled trial. The Lancet. 2004 2004/10/16/;364(9443):1411-1420. doi:<https://doi.org/10.1016/S0140-6736(04)17223-1>.

25. McCarthy JS, Marjason J, Elliott S, Fahey P, Bang G, Malkin E, Tierney E, Aked-Hurditch H, Adda C, Cross N, Richards JS, Fowkes FJI, Boyle MJ, Long C, Druilhe P, Beeson JG, Anders RF. A Phase 1 Trial of MSP2-C1, a Blood-Stage Malaria Vaccine Containing 2 Isoforms of MSP2 Formulated with Montanide® ISA 720. PLOS ONE. 2011;6(9):e24413. doi:10.1371/journal.pone.0024413.

26. Genton B, Betuela I, Felger I, Al-Yaman F, Anders RF, Saul A, Rare L, Baisor M, Lorry K, Brown GV, Pye D, Irving DO, Smith TA, Beck H-P, Alpers MP. A Recombinant Blood-Stage Malaria Vaccine Reduces *Plasmodium falciparum* Density and Exerts Selective Pressure on Parasite Populations in a Phase 1-2b Trial in Papua New Guinea. The Journal of Infectious Diseases. 2002;185(6):820-827. doi:10.1086/339342 %J The Journal of Infectious Diseases.

27. Dent AE, Nakajima R, Liang L, Baum E, Moormann AM, Sumba PO, Vulule J, Babineau D, Randall A, Davies DH, Felgner PL, Kazura JW. Plasmodium falciparum Protein Microarray Antibody Profiles Correlate With Protection From Symptomatic Malaria in Kenya. The Journal of Infectious Diseases. 2015;212(9):1429-1438. doi:10.1093/infdis/jiv224.

28. Rono J, Osier FHA, Olsson D, Montgomery S, Mhoja L, Rooth I, Marsh K, Färnert A. Breadth of Anti-Merozoite Antibody Responses Is Associated With the Genetic Diversity of Asymptomatic Plasmodium falciparum Infections and Protection Against Clinical Malaria. Clinical Infectious Diseases. 2013;57(10):1409-1416. doi:10.1093/cid/cit556.

29. Feng G, Kurtovic L, Agius PA, Aitken EH, Sacarlal J, Wines BD, Hogarth PM, Rogerson SJ, Fowkes FJI, Dobaño C, Beeson JG. Induction, decay, and determinants of functional antibodies following vaccination with the RTS,S malaria vaccine in young children. BMC Medicine. 2022 2022/08/25;20(1):289. doi:10.1186/s12916-022-02466-2.

30. Reiling L, Boyle MJ, White MT, Wilson DW, Feng G, Weaver R, Opi DH, Persson KEM, Richards JS, Siba PM, Fowkes FJI, Takashima E, Tsuboi T, Mueller I, Beeson JG. Targets of complement-fixing antibodies in protective immunity against malaria in children. Nature Communications. 2019 2019/02/05;10(1):610. doi:10.1038/s41467-019-08528-z.

31. Harris AW, Kurtovic L, Nogueira J, Bouzas I, Opi DH, Wines BD, Lee WS, Hogarth PM, Poumbourios P, Drummer HE, Valim C, Porto LC, Beeson JG. Induction of Fc-dependent functional antibodies against different variants of SARS-CoV-2 varies by vaccine type and prior infection. Commun Med (Lond). 2024 Dec 19;4(1):273. Epub 2024/12/20. doi:10.1038/s43856-024-00686-6. Cited in: Pubmed; PMID 39702507.

32. Feng G, Boyle MJ, Cross N, Chan J-A, Reiling L, Osier F, Stanisic DI, Mueller I, Anders RF, McCarthy JS. Human immunization with a polymorphic malaria vaccine candidate induced antibodies to conserved epitopes that promote functional antibodies to multiple parasite strains. J Infect Dis. 2018 Jul 1;218(1):35-43. English. doi:10.1093/infdis/jiy170. Cited in: Pubmed; PMID WOS:000434930900006.

33. Persson KE, Horton JL, Kurtovic L, McCarthy JS, Anders RF, Beeson JG. Declining antibody affinity over time after human vaccination with a Plasmodium falciparum merozoite vaccine candidate. The Journal of Infectious Diseases. 2024:jiae259.

34. Reddy SB, Anders RF, Beeson JG, Färnert A, Kironde F, Berenzon SK, Wahlgren M, Linse S, Persson KEM. High Affinity Antibodies to *Plasmodium falciparum* Merozoite Antigens Are Associated with Protection from Malaria. PLOS ONE. 2012;7(2):e32242. doi:10.1371/journal.pone.0032242.

35. RstudioTeam. RStudio: Integrated Development for R. Boston, MA: RStudio, PBC; 2020.

36. Dennison SM, Reichartz M, Seaton KE, Dutta S, Wille-Reece U, Hill AV, Ewer KJ, Rountree W, Sarzotti-Kelsoe M, Ozaki DA. Qualified Biolayer Interferometry Avidity Measurements Distinguish the Heterogeneity of Antibody Interactions with *Plasmodium falciparum* Circumsporozoite Protein Antigens. The Journal of Immunology. 2018;201(4):1315-1326.

37. Diebolder CA, Beurskens FJ, de Jong RN, Koning RI, Strumane K, Lindorfer MA, Voorhorst M, Ugurlar D, Rosati S, Heck AJ, van de Winkel JG, Wilson IA, Koster AJ, Taylor RP, Saphire EO, Burton DR, Schuurman J, Gros P, Parren PW. Complement is activated by IgG hexamers assembled at the cell surface. Science. 2014 Mar 14;343(6176):1260-3. Epub 2014/03/15. doi:10.1126/science.1248943. Cited in: Pubmed; PMID 24626930.

38. Vidarsson G, Dekkers G, Rispens T. IgG subclasses and allotypes: from structure to effector functions. Front Immunol. 2014;5:520. Epub 2014/11/05. doi:10.3389/fimmu.2014.00520. Cited in: Pubmed; PMID 25368619.

39. Tijani MK, Reddy SB, Langer C, Beeson JG, Wahlgren M, Nwuba RI, Persson KEM. Factors influencing the induction of high affinity antibodies *to Plasmodium falciparum* merozoite antigens and how affinity changes over time. Scientific Reports. 2018 2018/06/13;8(1):9026. doi:10.1038/s41598-018-27361-w.

40. Akpogheneta O, Dunyo S, Pinder M, Conway D. Boosting antibody responses to *Plasmodium falciparum* merozoite antigens in children with highly seasonal exposure to infection. Journal of Parasite Immunology. 2010;32(4):296-304.

41. Wu RL, Idris AH, Berkowitz NM, Happe M, Gaudinski MR, Buettner C, Strom L, Awan SF, Holman LA, Mendoza F, Gordon IJ, Hu Z, Campos Chagas A, Wang LT, Da Silva Pereira L, Francica JR, Kisalu NK, Flynn BJ, Shi W, Kong WP, O'Connell S, Plummer SH, Beck A, McDermott A, Narpala SR, Serebryannyy L, Castro M, Silva R, Imam M, Pittman I, Hickman SP, McDougal AJ, Lukoskie AE, Murphy JR, Gall JG, Carlton K, Morgan P, Seo E, Stein JA, Vazquez S, Telscher S, Capparelli EV, Coates EE, Mascola JR, Ledgerwood JE, Dropulic LK, Seder RA, Team VRCS. Low-Dose Subcutaneous or Intravenous Monoclonal Antibody to Prevent Malaria. N Engl J Med. 2022 Aug 4;387(5):397-407. Epub 2022/08/04. doi:10.1056/NEJMoa2203067. Cited in: Pubmed; PMID 35921449.

42. Williams KL, Guerrero S, Flores-Garcia Y, Kim D, Williamson KS, Siska C, Smidt P, Jepson SZ, Li K, Dennison SM, Mathis-Torres S, Chen X, Wille-Reece U, MacGill RS, Walker M, Jongert E, King CR, Ockenhouse C, Glanville J, Moon JE, Regules JA, Tan YC, Cavet G, Lippow SM, Robinson WH, Dutta S, Tomaras GD, Zavala F, Ketchem RR, Emerling DE. A candidate antibody drug for prevention of malaria. Nat Med. 2024 Jan;30(1):117-129. eng. W.H.R. owns equity in, serves as a consultant to and is a member of the Board of Directors of Atreca, Inc. K.L.W., S.G., K.S.W. and S.M.L. own equity in, and are employed by, Atreca, Inc. D.E.E. and M.W. own equity in, and serve as consultants to, Atreca, Inc. G.C. and D.K. own equity in Atreca, Inc. C.S., P.S., S.Z.J. and R.R.K. are employed by Just – Evotec Biologics. E.J. owns equity in, and is employed by, GSK. U.W.-R. is employed by BioNTech. J.G. owns equity in, is employed by and is a member of the Board of Directors of Centivax, Inc. Y.C.T. owns equity in Atreca, Inc. and is employed by Nuevocor Pte. Ltd. K.L.W., S.M.L., R.R.K. and D.E.E. are coinventors on patent applications filed by Atreca, Inc. that include antibodies to CSP. The remaining authors declare no competing interests. Epub 20240102. doi:10.1038/s41591-023-02659-z. Cited in: Pubmed; PMID 38167935.

43. Beeson JG, Drew DR, Boyle MJ, Feng G, Fowkes FJ, Richards JS. Merozoite surface proteins in red blood cell invasion, immunity and vaccines against malaria. FEMS Microbiol Rev. 2016 May;40(3):343-72. Epub 2016/02/03. doi:10.1093/femsre/fuw001. Cited in: Pubmed; PMID 26833236.

44. Bellamy DG. Investigating protective mechanisms of anti-sporozoite vaccines. <https://ora.ox.ac.uk/objects/uuid:0233a217-674b-4672-8ae3-056053ecf207>: Oxford University; 2023. PhD Thesis. <https://ora.ox.ac.uk/objects/uuid:0233a217-674b-4672-8ae3-056053ecf207>.

45. Palm A-KE, Henry C. Remembrance of Things Past: Long-Term B Cell Memory After Infection and Vaccination [Review]. Frontiers in Immunology. 2019 2019-July-31;10:1787. English. doi:10.3389/fimmu.2019.01787.

46. Adda CG, MacRaild CA, Reiling L, Wycherley K, Boyle MJ, Kienzle V, Masendycz P, Foley M, Beeson JG, Norton RS, Anders RF. Antigenic characterization of an intrinsically unstructured protein, Plasmodium falciparum merozoite surface protein 2. Infect Immun. 2012 Dec;80(12):4177-85. eng. Epub 2012/09/12. doi:10.1128/iai.00665-12. Cited in: Pubmed; PMID 22966050.

47. Reddy SB, Anders RF, Cross N, Mueller I, Senn N, Stanisic DI, Siba PM, Wahlgren M, Kironde F, Beeson JG, Persson KEM. Differences in affinity of monoclonal and naturally acquired polyclonal antibodies against *Plasmodium falciparum* merozoite antigens. BMC Microbiology. 2015 Jul 3;15(1):133. Epub 2015/07/08. doi:10.1186/s12866-015-0461-1. Cited in: Pubmed; PMID 26149471.
